# Low-power photodynamic therapy induces survival signaling in perihilar cholangiocarcinoma cells

**DOI:** 10.1186/s12885-015-1994-2

**Published:** 2015-12-26

**Authors:** Ruud Weijer, Mans Broekgaarden, Rowan F. van Golen, Esther Bulle, Esther Nieuwenhuis, Aldo Jongejan, Perry D. Moerland, Antoine H. C. van Kampen, Thomas M. van Gulik, Michal Heger

**Affiliations:** Department of Experimental Surgery, Academic Medical Center, University of Amsterdam, Meibergdreef 9, 1105 AZ Amsterdam, The Netherlands; Bioinformatics Laboratory, Academic Medical Center, University of Amsterdam, Meibergdreef 9, 1105 AZ Amsterdam, The Netherlands

**Keywords:** Drug delivery system, Metallated phthalocyanines, Non-resectable perihilar cholangiocarcinoma, Therapeutic recalcitrance, Tumor targeting

## Abstract

**Background:**

Photodynamic therapy (PDT) of solid cancers comprises the administration of a photosensitizer followed by illumination of the photosensitizer-replete tumor with laser light. This induces a state of local oxidative stress, culminating in the destruction of tumor tissue and microvasculature and induction of an anti-tumor immune response. However, some tumor types, including perihilar cholangiocarcinoma, are relatively refractory to PDT, which may be attributable to the activation of survival pathways in tumor cells following PDT (*i.e.*, activator protein 1 (AP-1)-, nuclear factor of kappa light polypeptide gene enhancer in B-cells (NF-κB)-, hypoxia-inducible factor 1-alpha (HIF-1α)-, nuclear factor (erythroid-derived 2)-like 2 (NFE2L2)-, and unfolded protein response-mediated pathways).

**Methods:**

To assess the activation of survival pathways after PDT, human perihilar cholangiocarcinoma (SK-ChA-1) cells were subjected to PDT with zinc phthalocyanine (ZnPC)-encapsulating liposomes. Following 30-minute incubation with liposomes, the cells were either left untreated or treated at low (50 mW) or high (500 mW) laser power (cumulative light dose of 15 J/cm^2^). Cells were harvested 90 min post-PDT and whole genome expression analysis was performed using Illumina HumanHT-12 v4 expression beadchips. The data were interpreted in the context of the survival pathways. In addition, the safety of ZnPC-encapsulating liposomes was tested both *in vitro* and *in vivo*.

**Results:**

PDT-treated SK-ChA-1 cells exhibited activation of the hypoxia-induced stress response via HIF-1α and initiation of the pro-inflammatory response via NF-кB. PDT at low laser power in particular caused extensive survival signaling, as evidenced by the significant upregulation of HIF-1- (*P* < 0.001) and NF-кB-related (*P* < 0.001) genes. Low-power PDT was less lethal to SK-ChA-1 cells 90 min post-PDT, confirmed by annexin V/propidium iodide staining. *In vitro* toxicogenomics and toxicological testing in chicken embryos and mice revealed that the ZnPC-encapsulating liposomes are non-toxic.

**Conclusions:**

PDT-treated perihilar cholangiocarcinoma cells exhibit extensive survival signaling that may translate to a suboptimal therapeutic response and possibly tumor recurrence. These findings encourage the development of photosensitizer delivery systems with co-encapsulated inhibitors of survival pathways.

**Electronic supplementary material:**

The online version of this article (doi:10.1186/s12885-015-1994-2) contains supplementary material, which is available to authorized users.

## Background

Photodynamic therapy (PDT) is a non-to-minimally invasive treatment modality that is used for the curative or palliative treatment of early-stage and late-stage solid cancers, respectively. The therapy relies on the accumulation of a non-toxic photosensitizer in the tumor following topical or systemic administration. Subsequently, the tumor is light-irradiated locally at a wavelength that corresponds to the red absorption peak of the photosensitizer. This leads to photosensitizer activation and generation of cytotoxic reactive oxygen species (ROS) via type I (superoxide anion) and/or type II (singlet oxygen) photochemical reactions. Extensive intratumoral ROS production initiates several key processes that culminate in the removal of the tumor, including: (1) induction of different forms of tumor cell death, (2) destruction of tumor microvasculature, (3) blood flow stasis and consequent tumor hypoxia/anoxia, and (4) induction of an anti-tumor immune response (reviewed in [1–3]).

Although PDT is highly effective in some cancer types (*e.g.*, basal cell carcinoma, early-stage esophageal carcinoma) [4–7], other solid cancers are relatively unresponsive to PDT (*e.g*., nasopharyngeal carcinoma [8], perihilar cholangiocarcinoma [9]). This therapeutic recalcitrance may be explained by three key factors. First, the route of photosensitizer administration may be suboptimal for a specific tumor type, thereby deterring optimal photosensitizer accumulation in the tumor. Second, the approved first-generation photosensitizers (*i.e.*, hematoporphyrin derivatives and 5-aminolevulinic acid) exhibit poor photophysical and physicochemical properties, leading to insufficient and/or heterogeneous ROS production throughout the tumor bulk. Third, activation of survival pathways by tumor cells as a result of PDT may lead to insufficient tumor cell death following PDT [10].

To resolve these issues, a novel PDT modality was proposed based on the encapsulation of the photosensitizer zinc phthalocyanine (ZnPC) into polyethylene glycol (PEG)-coated liposomes [11]. Accordingly, three distinct ZnPC-containing nanoparticulate formulations were developed that are either targeted to tumor cells (tumor cell-targeting liposomes), tumor endothelium (endothelium-targeting liposomes), or tumor interstitium (interstitially-targeted liposomes (ITLs)) [11]. This comprehensive tumor-targeting strategy is expected to augment therapeutic efficacy and minimize photosensitivity and phototoxicity that are observed in patients treated with currently approved PDT modalities.

**Fig. 1:**
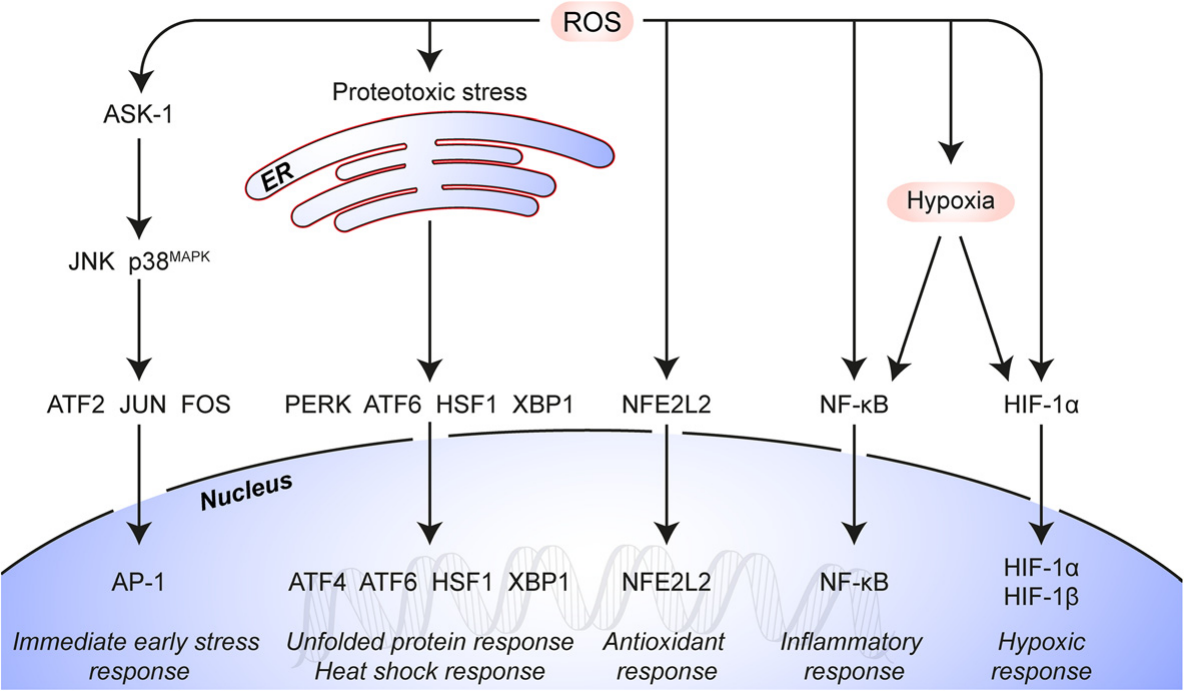
Induction of survival signaling after PDT. PDT-mediated ROS production results in activation of (1) the immediate early gene response via activator protein 1 (AP-1), (2) the unfolded protein response in reaction to endoplasmic reticulum (ER) stress, (3) the antioxidant response via nuclear factor (erythroid-derived 2)-like 2 (NFE2L2), (4) the inflammatory response via activation of nuclear factor of kappa light polypeptide gene enhancer in B-cells (NF-кB), and (5) the hypoxia-induced stress response via hypoxia-inducible factor 1-alpha (HIF-1α). Data and figure adapted from [10]

The aims of this study were to evaluate whether ZnPC-encapsulating ITLs (ZnPC-ITLs) are safe for future clinical application using toxicogenomics [12], chicken embryos, and mice as toxicological test models and to study the PDT-induced activation of survival pathways in human perihilar cholangiocarcinoma cells – *i.e.*, cells derived from a cancer that is recalcitrant to PDT. For the latter aim, whole genome gene expression profiles were determined in the early phase (90 min) after PDT in accordance with literature [13] and the data were analyzed in the context of the five major PDT-induced survival pathways [10] (Fig. 1).

The main findings were, first, that ZnPC-ITLs are not toxic *in vitro* and *in vivo* up to a 500-μM and 2.5-mM final lipid concentration, respectively, at a ZnPC:lipid molar ratio of 0.003. Second, irradiation of cells at low laser power (50 mW, 15 J/cm^2^) caused considerable survival signaling after PDT via activation of hypoxia-inducible factor 1 (HIF-1) and nuclear factor of kappa light polypeptide gene enhancer in B-cells (NF-кB), which was associated with limited photokilling capacity. Irradiation of cells at high laser power (500 mW, 15 J/cm^2^) was associated with less extensive survival signaling and resulted in more profound cell death.

## Results

### PDT efficacy

**Fig. 2:**
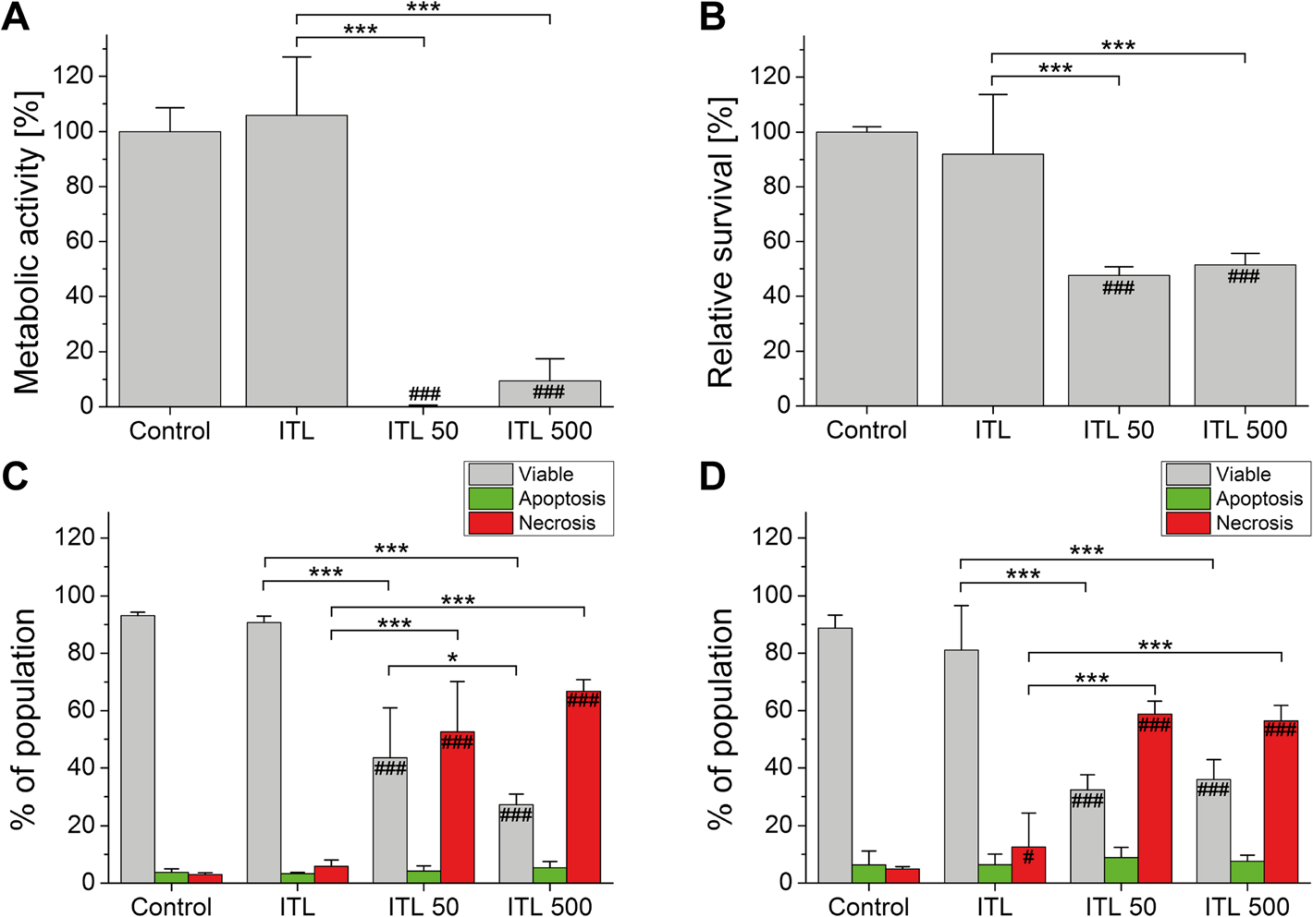
Viability of SK-ChA-1 cells after ZnPC-ITL-PDT. SK-ChA-1 cells were incubated with 500 μM ZnPC-ITLs (final lipid concentration) and kept in the dark (ITL) or were irradiated with 50-mW (ITL 50) or 500-mW (ITL 500) laser light at a cumulative radiant exposure of 15 J/cm^2^. Metabolic activity and the extent of cell death were assessed after 24 hours with a WST-1 assay (**a**) and SRB assay (**b**), respectively. Data were normalized to control cells that were set at 100 %. Alternatively, the mode of cell death was assessed 90 minutes (**c**) or 24 hours (**d**) post-PDT by flow cytometry. For this purpose, cells were stained with Alexa Fluor 488-conjugated annexin V and propidium iodide (PI). Necrotic cells are represented in red (PI-positive), apoptotic cells are shown in green (PI-negative, annexin V-positive), and healthy cells are represented in white (PI-negative, annexin V-negative). Values are presented as mean + SD for *n* = 6 per group. Readers are referred to section Statistical analysis for the significance of the statistical symbols

The *in vitro* proof-of-concept regarding ZnPC-ITLs as part of a novel multi-targeting strategy for PDT was provided previously [14]. However, this study did not examine the effect of laser power on post-PDT viability. It was hypothesized that low laser power (*i.e*., low degree of ROS production per unit time) would allow cells to cope with ROS-induced damage, whereas high laser power would be more toxic to cells. To investigate the influence of laser power on PDT efficacy, SK-ChA-1 cells were incubated with ZnPC-ITLs and either not irradiated (dark toxicity, designated as ‘ITL’) or irradiated at high laser power (500 mW, designated as ‘ITL 500’) or low laser power (50 mW, designated as ‘ITL 50’) with a cumulative radiant exposure of 15 J/cm^2^. As shown in Fig. 2a, cells in the ITL group exhibited slightly higher metabolic activity than the control cells 24 h after treatment, whereas metabolic activity was completely abrogated in the treated cells. Cell death was assessed with the SRB protein assay, which revealed that cell viability had decreased to 47.6 % and 51.4 % (normalized to the control group) in the ITL 50 and ITL 500 groups, respectively (Fig. 2b).

In addition, SK-ChA-1 cells were stained with annexin V and PI 90 min (Fig. 2c) and 24 h (Fig. 2d) after PDT to evaluate the mode of cell death by flow cytometry. After 90 min, the ITL group paralleled the control group with a cell viability of approximately 90 %, whereas 43.6 % and 27.2 % of cells were viable in the ITL 50 and ITL 500 groups, respectively (Fig. 2c). The difference in cell viability between the treatment groups was abolished 24 h after PDT (Fig. 2d), suggesting that execution of cell death programs had not yet completed 90 min after low light dose PDT and that laser power therefore dictates the rate at which cell death programs are executed.

### In vitro and in vivo toxicity

**Fig. 3:**
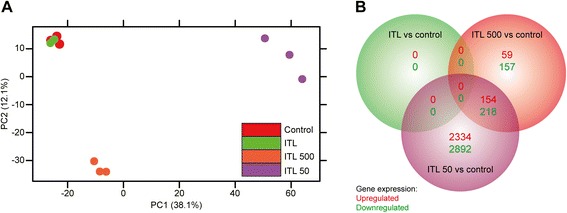
**a** Principal component analysis of SK-ChA-1 cells that were either untreated (in red), incubated with 500 μM ZnPC-ITLs (final lipid concentration) and kept in the dark (ITL, in green), or treated with 500-mW (ITL 500, in orange) or 50-mW (ITL 50, in purple) laser light. The extent to which a principal component (PC) accounts for the variability in the data is indicated in parentheses. **b** Venn diagram showing the number of non-overlapping upregulated and downregulated genes per group (in-circle values) and the number of overlapping upregulated and downregulated genes (values in the respective overlapping region) between the various treatment groups compared to the control group. The total number of genes that were up- and downregulated per group comprises the sum of all regions in a given circle

Next to efficacy, the safety of a new liposomal formulation is a critical parameter in the preclinical development trajectory. Therefore, *in vivo* toxicity was evaluated in two different animal models, namely in chicken embryos and in C57BL/6 mice. The chicken embryo model was chosen to assess acute toxicity, as it is a cheap and suitable substitute for mammalian models [15]. Alternatively, a mouse model was used to study long-term toxicity. As shown in Additional file 1: Figure S1, systemically administered ZnPC-ITLs did not exhibit any toxicity. In addition, whole genome microarray-based toxicogenomics is considered a valuable tool for evaluating the toxicity of xenobiotics [12, 16]. Therefore, as a complementary method to the *in vivo* toxicity testing, the *in vitro* toxicity of ZnPC-ITLs was analyzed in SK-ChA-1 cells by microarray analysis. SK-ChA-1 control cells and cells that were incubated with ZnPC-ITLs in the dark (ITL) exhibited similar transcriptional responses (Fig. 3a). None of the genes were differentially expressed when comparing the ITL group to the control group, corroborating the *in vivo* data at a molecular level.

### Gross transcriptional response to PDT

In addition to the toxicogenomic profile of ZnPC-ITLs, the transcriptomic data was used to gain insight in the immediate early gene response [13] and explain the differences in cell viability that were observed 90 min post-PDT (Fig. 2c). As depicted in Fig. 3a, the global molecular response of the ITL 50 and ITL 500 groups were not associated and both groups showed a distinct response relative to the control group. The ITL 500 modality resulted in the upregulation of 213 genes and downregulation of 375 genes (588 total) compared to the control regimen (Fig. 3b). The number of differentially expressed genes in the ITL 50 group relative to control was ~10-fold greater (*i.e.*, 5,598) versus the ITL 500 group. Cells in the ITL 50 and the ITL 500 group exhibited some overlap in differentially expressed genes, namely 154 upregulated genes and 218 downregulated genes.

### Differential gene regulation in response to PDT

**Fig. 4:**
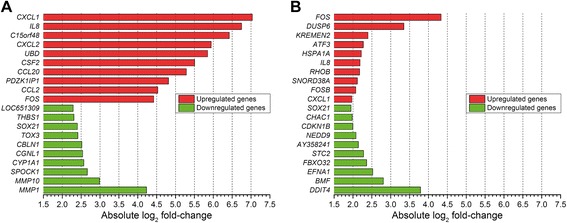
Top upregulated and downregulated PDT-induced genes as expressed by the log_2_ fold-change in gene expression in the ITL 50 (**a**) and ITL 500 group (**b**) compared to the control group. Genes were ordered by decreasing absolute log_2_ fold-change. Values are presented as the mean of *n* = 3 per group

To gain insight in the key processes that are initiated by PDT at the molecular level, the top increased and decreased genes were ranked based on the log_2_ fold-change (Fig. 4, with more detailed information in Tables 1 and 2). Compared to the control group, the ITL 50 group exhibited more profound changes in gene expression than the ITL 500 group. Of note, the microarray expression data were validated by determining the transcript levels of specific genes by qRT-PCR, which revealed a strong correlation (Additional file 2: Figure S2).

**Table 1:** Top 10 most up- and downregulated genes induced by the 50-mW PDT regimen

Gene:	Full name:	Function:
*CXCL1*	chemokine (C-X-C motif) ligand 1	Cell proliferation, chemotaxis, inflammation
*IL8*	interleukin 8	Angiogenesis, chemotaxis, inflammation
*C15orf48*	chromosome 15 open reading frame 48	
*CXCL2*	chemokine (C-X-C motif) ligand 2	Chemotaxis, inflammation
*UBD*	ubiquitin D	Protein ubiquitination, aggresome formation, myeloid DC differentiation
*CSF2*	colony stimulating factor 2	Macrophage activation, DC differentiation, immune response
*CCL20*	chemokine (C-C motif) ligand 20	Chemotaxis, inflammation
*PDZK1IP1*	PDZK1 interacting protein 1	
*CCL2*	chemokine (C-C motif) ligand 2	Chemotaxis, inflammation
*FOS*	FBJ murine osteosarcoma viral oncogene homolog	Inflammation, cellular response to ROS, DNA methylation
*LOC651309*		
*THBS1*	thrombospondin 1	Cell adhesion, extracellular matrix organization
*SOX21*	SRY (sex determining region Y)-box 21	Transcription from RNA polymerase II promoter, SC differentiation
*TOX3*	TOX high mobility group box family member 3	DNA-dependent transcription
*CBLN1*	cerebellin 1 precursor	Positive regulation of synapse assembly
*CGNL1*	cingulin-like 1	Motor activity
*CYP1A1*	cytochrome P450, family 1, subfamily A, polypeptide 1	Xenobiotic metabolic processes, drug metabolic processes
*SPOCK1*	testican 1	Neuron migration, neurogenesis
*MMP10*	matrix metallopeptidase 10	Proteolysis, extracellular matrix disassembly
*MMP1*	matrix metallopeptidase 1	Proteolysis, extracellular matrix disassembly

Gene functions were obtained from The Gene Ontology Consortium (http://www.geneontology.org/)

**Table 2:** Top 10 most up- and downregulated genes induced by the 500-mW PDT regimen

Gene:	Full name:	Function:
*FOS*	FBJ murine osteosarcoma viral oncogene homolog	Inflammation, cellular response to ROS, DNA methylation
*DUSP6*	dual specificity phosphatase 6	Inactivation of MAPK activity
*KREMEN2*	kringle containing transmembrane protein 2	Wnt receptor signaling pathway
*ATF3*	activating transcription factor 3	Response to stress
*HSPA1A*	heat shock 70 kDa protein 1A	Response to unfolded protein, ubiquitin protein ligase binding
*IL8*	interleukin 8	Angiogenesis, chemotaxis, inflammation
*RHOB*	ras homolog family member B	GTP binding, apoptotic process, cellular response to H_2_O_2_
*SNORD38A*	small nucleolar RNA, C/D box 38A	
*FOSB*	FBJ murine osteosarcoma viral oncogene homolog B	Transcription factor binding
*CXCL1*	chemokine (C-X-C motif) ligand 1	Cell proliferation, chemotaxis, inflammation
*SOX21*	SRY (sex determining region Y)-box 21	Transcription from RNA polymerase II promoter, stem cell differentiation
*CHAC1*	ChaC, cation transport regulator homolog 1	Intrinsic apoptotic signaling pathway in response to ER stress, negative regulator of Notch signaling pathway
*CDKN1B*	cyclin-dependent kinase inhibitor 1B	Cell cycle arrest
*NEDD9*	neural precursor cell expressed, developmentally down-regulated 9	Cell adhesion, cell division, cytoskeleton organization
*AY358241*		
*STC2*	stanniocalcin 2	Cellular calcium ion homeostasis
*FBXO32*	F-box protein 32	Protein ubiquitination, response to denervation involved in regulation of muscle adaptation
*EFNA1*	ephrin-A1	Activation of MAPK activity, cell migration, aortic valve morphogenesis
*BMF*	Bcl2 modifying factor	Positive regulation of intrinsic apoptotic signaling pathway
*DDIT4*	DNA-damage-inducible transcript 4	Response to hypoxia, intrinsic apoptotic signaling pathway in response to DNA damage by p53 class mediator, negative regulation of TOR signaling cascade

Gene functions were obtained from The Gene Ontology Consortium (http://www.geneontology.org/)

As shown in Table 1, the top upregulated genes in the ITL 50 group are involved in chemotaxis (chemokine (C-X-C motif) ligand 1 (*CXCL1*) and *CXCL2*), inflammation (interleukin 8 (*IL8*), FBJ murine osteosarcoma viral oncogene homolog (*FOS*)), and the immune response (colony stimulating factor 2 (*CSF2*)), whereas downregulated genes are associated with cell adhesion (thrombospondin 1 (*THBS1*)) and the extracellular matrix (matrix metallopeptidase 1 (*MMP1*), *MMP10*). In contrast, treatment of SK-ChA-1 cells at 500-mW laser power activated transcripts related to mitogen-activated protein (MAP) kinase (MAPK) signaling (dual specificity phosphatase 6 (*DUSP6*)), the stress response (activating transcription factor 3 (*ATF3*)), and response to ROS and unfolded proteins (*FOS*, heat shock 70 kDa protein 1A (*HSPA1A*)) (Table 2). High-power irradiation also resulted in downregulation of genes involved in cell cycle arrest (cyclin-dependent kinase inhibitor 1B (*CDKN1B*)) and apoptosis initiation (Bcl2 modifying factor (*BMF*)).

In addition, gene ontology analysis was performed using the DAVID Bioinformatics Resources 6.7 database to gain insight in the upregulated genes (absolute log_2_ fold-change of > 1, corrected P-value of < 0.05 (section Data analysis and processing)) in the ITL 50 and ITL 500 group in terms of biological processes. Overrepresented gene ontology (GO) terms were evaluated and are presented in Table 3. Characterization of the top 8 overrepresented GO terms revealed that the GO term “response to stress” applied to both the ITL 50 (involving 92 genes) and the ITL 500 (involving 16 genes) group. In the ITL 50 group, genes annotated with the GO terms “response to biotic stimulus” and “apoptosis” were overrepresented, as reflected by a *P*-value of 1.2 × 10^−17^ and 2.0 × 10^−7^, respectively. In contrast, the GO terms “MAP kinase phosphatase” and “regulation of cellular process” were overrepresented in the ITL 500 group. In summary, the main processes that were initiated following PDT include oxidative stress, cell death, and inflammation.

**Table 3:** Overrepresented GO terms in the ITL 50 and ITL 500 group

ITL 50			
Cluster [ES]	GO term	Count	*P*-value (FDR)
1 [12.96]	Response to biotic stimulus	45	1.2E-17
2 [11.83]	Response to stress	92	6.2E-13
3 [5.89]	Apoptosis	39	2.0E-7
4 [5.77]	Response to cytokine stimulus	14	9.8E-8
5 [5.13]	Regulation of apoptosis	47	2.4E-7
6 [4.92]	Regulation of response to stress	25	1.3E-7
7 [4.38]	Chemotaxis	19	1.3E-7
8 [4.18]	bZIP transcription factor	10	5.7E-6
ITL 500			
Cluster [ES]	GO term	Count	*P*-value
1 [3.97]	Response to stress	16	2.4E-5
2 [3.55]	MAP kinase phosphatase	4	1.8E-6
3 [3.15]	Regulation of cellular process	32	7.2E-5
4 [2.68]	Response to chemical stimulus	12	6.1E-4
5 [2.41]	Angiogenesis	6	5.7E-5
6 [2.33]	Fos transforming protein	4	8.5E-7
7 [2.08]	Regulation of cell proliferation	8	6.1E-3
8 [1.89]	Regulation of catalytic activity	9	2.2E-3

Gene ontology (GO) analysis was performed using the DAVID Bioinformatics Resources 6.7 database (https://david.ncifcrf.gov/). Significantly upregulated genes were loaded into DAVID and the HumanHT-12_V3_0_R2_11283641_A was selected as a background reference. An EASE score of 0.1 was used in the analysis and FDR-corrected *P*-values are presented

### Activation of survival pathways following PDT

**Fig. 5:**
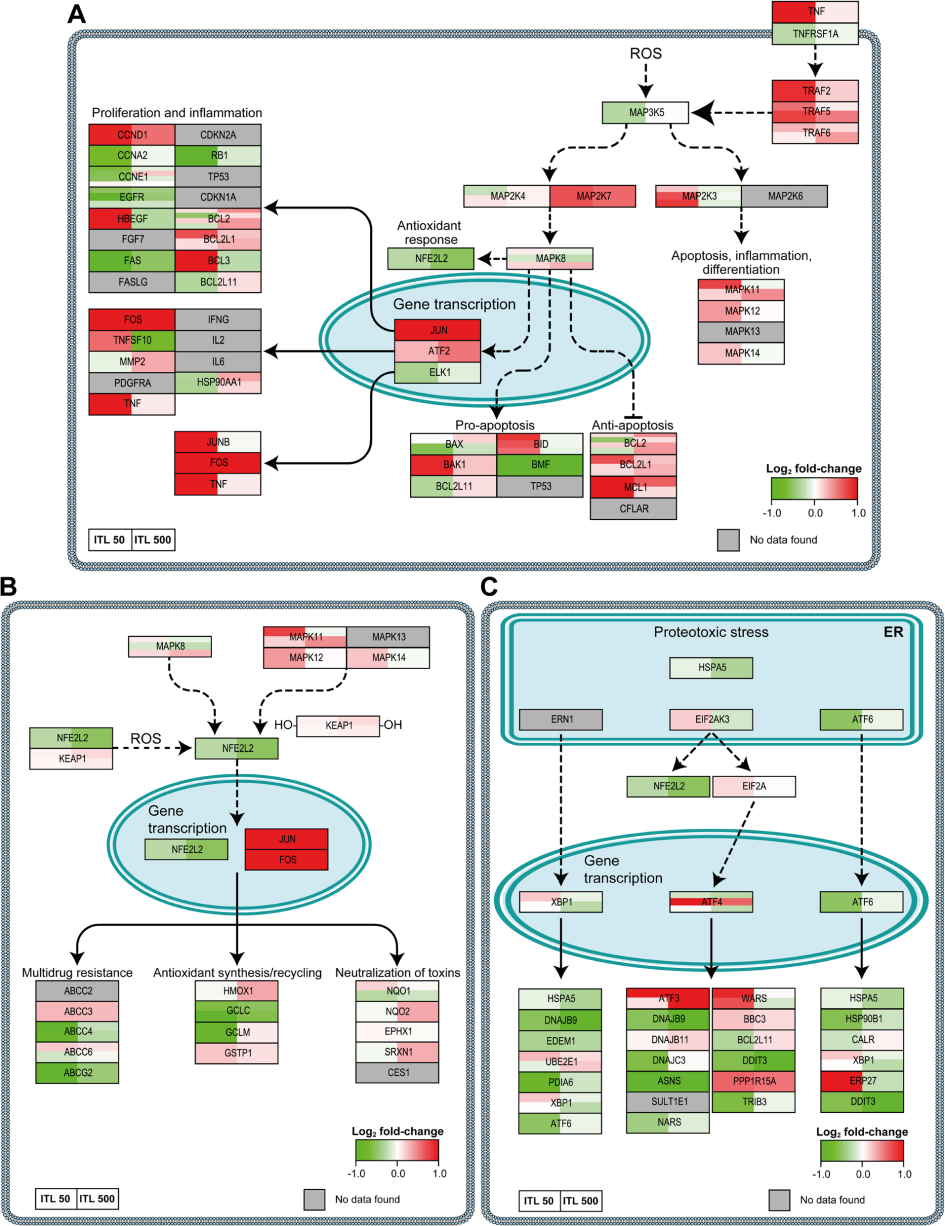
PDT-induced survival signaling. Genes are mapped that are involved in MAP3K5 (also known as ASK-1) signaling (**a**), NFE2L2 signaling (**b**), and the unfolded protein response following PDT (**c**). The color and intensity of the box indicates the direction and extent of the log_2_ fold-change for the indicated gene, respectively (lower right corner of each panel). Grey boxes signify probes that exhibited poor quality or were not included in the gene expression analysis. Each gene box, which typically comprises multiple probes as indicated by vertical splits, is horizontally divided in two halves corresponding to the PDT regimens (legend lower left). All comparisons were made between the PDT-treated groups versus the control group. Dashed lines indicate interactions that are not directly transcriptionally regulated. The molecular pathways were adapted from [10]

**Fig. 6:**
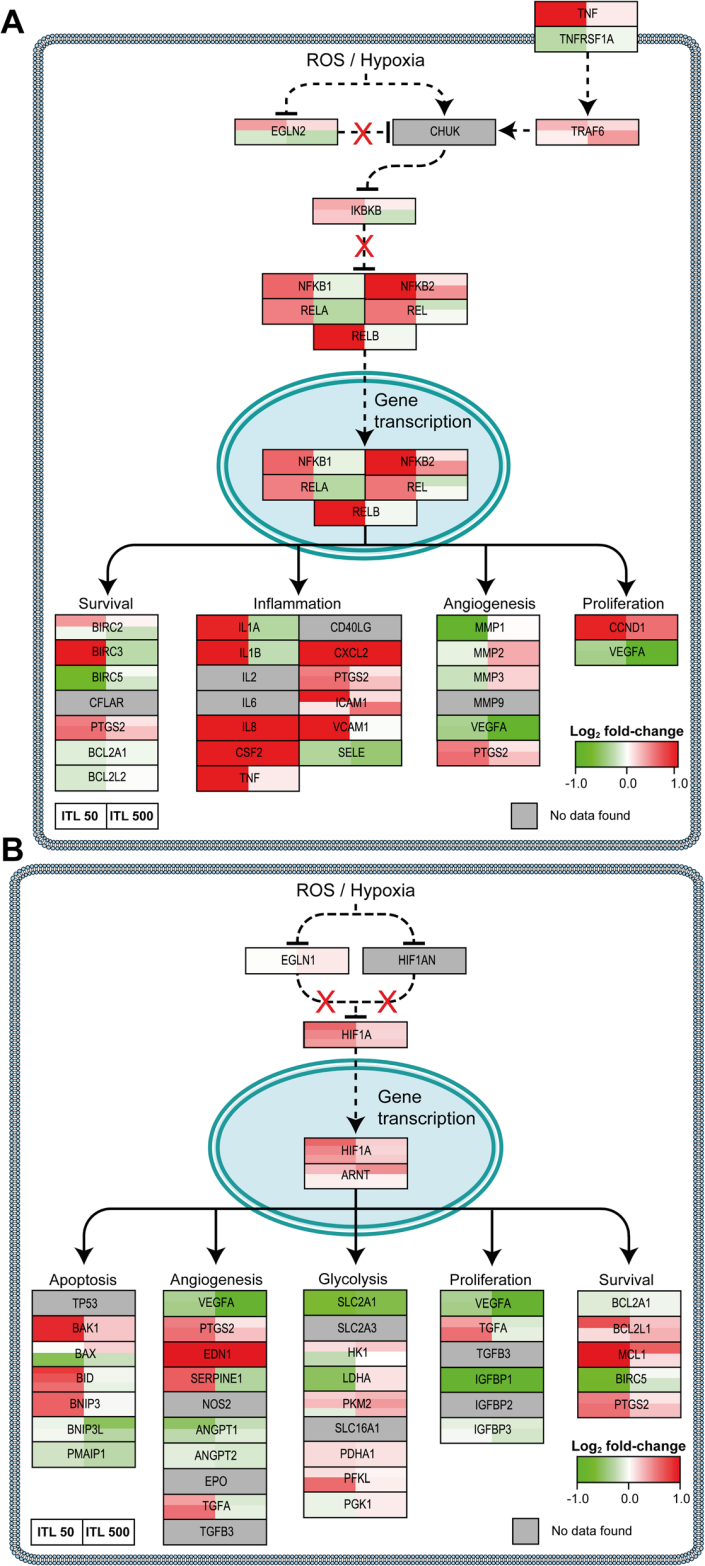
PDT-induced survival signaling. Genes are mapped that are involved in NF-кB signaling (**a**) and HIF-1 signaling (**b**) following PDT. The color and intensity of the box indicates the direction and extent of the log_2_ fold-change for the indicated gene, respectively (lower right corner of each panel). Grey boxes signify probes that exhibited poor quality or were not included in the gene expression analysis. Each gene box, which typically comprises multiple probes as indicated by vertical splits, is horizontally divided in two halves corresponding to the PDT regimens (legend lower left). All comparisons were made between the PDT-treated groups versus the control group. Dashed lines indicate interactions that are not directly transcriptionally regulated. The molecular pathways were adapted from [10]

Based on the survival pathways depicted in Fig. 1 and described in [10], survival signaling pathways were constructed in PathVisio to investigate whether and to what extent the PDT modalities initiated survival signaling in SK-ChA-1 cells (Figs. 5 and 6).

#### Activation of ASK-1 and consequent JNK and p38 MAPK signaling

Apoptosis signal regulating kinase 1 (ASK-1) can be activated in two distinct ways: (1) as a result of ROS production and (2) via tumor necrosis factor (TNF) production and subsequent tumor necrosis factor receptor-associated factor 2 (TRAF2), TRAF5, and TRAF6 signaling, all of which associate with ASK-1 and stimulate autophosphorylation and activation of ASK1 [17]. As depicted in Fig. 5a, activator protein 1 (AP-1, consisting of JUN and FOS, amongst others) was upregulated in both the ITL 50 and ITL 500 groups. Relative to the control group, numerous downstream genes of AP-1 signaling that exert pro-inflammatory and proliferative functions were more highly expressed in the ITL 50 group, including *TNF*, cyclin D1 (*CCND1*), heparin-binding EGF-like growth factor (*HBEGF*), B-cell CLL/lymphoma 3 (*BCL3*), and jun B proto-oncogene (*JUNB*) compared to the ITL 500 group.

#### NFE2L2 signaling following PDT

Nuclear factor (erythroid-derived 2)-like 2 (NFE2L2) is a transcription factor that is ubiquitously expressed in the cytoplasm and degraded via kelch-like ECH-associated protein 1 (KEAP1) under non-stressed conditions. During oxidative stress the redox-sensitive KEAP1 is oxidized, causing dissociation of KEAP1 from NFE2L2 [18]. In turn, NFE2L2 dimerizes with small Maf, JUN, and FOS proteins, which then translocate to the nucleus to bind to the antioxidant responsive element (ARE) [19, 20]. This binding initiates transcription of a plethora of antioxidant-responsive genes. An overview of the effects of ZnPC-ITL-PDT treatment on the NFE2L2-mediated pathway is shown in Fig. 5b. PDT resulted in modest downregulation of *NFE2L2* transcription levels, although cells in both the ITL 50 and ITL 500 groups upregulated NFE2L2 binding partners (*JUN*, *JUNB*, *FOS*). Despite the fact that *NFE2L2* was downregulated in the ITL 500 group, several NFE2L2 target genes were upregulated (*e.g.*, heme oxygenase 1 (*HMOX1*), NAD(P)H dehydrogenase, quinone 2 (*NQO2)*, sulfiredoxin 1 (*SRXN1*)). Furthermore, expression of various genes involved in glutathione and redox cycling (glutamate-cysteine ligase, catalytic subunit (*GCLC*), glutamate-cysteine ligase, modifier subunit (*GCLM*), glutathione reductase (*GSR*)) were decreased in the ITL 50 group. Overall, there was no unequivocal induction of NFE2L2-related antioxidant-responsive genes 90 min after PDT in either group (Table 4).

**Table 4:** A ROAST gene set test was performed to evaluate whether a specific survival pathway was either up- (▲) or downregulated (▼)

		ITL 50		ITL 500	
Geneset	Genes	Direction	*P*-value (FDR)	Direction	*P*-value (FDR)
AP-1	18	▲	0.102	▼	0.927
NFE2L2	13	▼	<0.001	▲	0.927
UPR	22	▼	0.141	▼	0.164
HIF-1	28	▲	<0.001	▼	0.059
NF-κB	21	▲	<0.001	▲	0.059

The gene sets are based on the specific survival pathways as presented in Figs. 5 and 6. FDR-corrected *P*-values are presented

#### Unfolded protein response following PDT

The unfolded protein response (UPR) is a process that is initiated upon ER stress. In response to the accumulation of unfolded and misfolded proteins in the ER, protein translation is stalled, unfolded and misfolded proteins are degraded, and molecular players involved in protein folding are upregulated (reviewed in [21]). However, apoptotic cell death is triggered when the amount of unfolded and misfolded proteins exceeds a certain threshold [21, 22]. During UPR signaling, HSPA5 binds unfolded and misfolded proteins in the ER lumen, which causes activation of endoplasmic reticulum to nucleus signaling 1 (ERN1), eukaryotic translation initiation factor 2-alpha kinase 3 (EIF2AK3), and ATF6 (Fig. 5c). Activation of these proteins in turn triggers the release of various transcription factors, which include X-box binding protein 1 (XBP1), ATF4, and ATF6. As shown in Fig. 5c, both the ITL 50 and ITL 500 groups did not clearly induce these transcription factors. In fact, various downstream genes of XBP1, ATF4, and ATF6 were downregulated rather than upregulated (*e.g.*, ER degradation enhancer, mannosidase alpha-like 1 (*EDEM1*), heat shock protein 90 kDa beta (*HSP90B1*)) in both groups 90 min after PDT, altogether attesting to the disinvolvement of the UPR survival pathway in ZnPC-ITL-PDT.

#### Activation of pro-inflammatory signaling via NF-кB

NF-кB consists of a subfamily of proteins that include NF-кB1, NF-кB2, v-rel avian reticuloendotheliosis viral oncogene homolog (REL), RELA, and RELB. Activation of NF-кB following PDT can occur by various pathways as shown in Fig. 6a. First, NF-кB can be activated via direct activation of conserved helix-loop-helix ubiquitous kinase (CHUK) during hypoxic conditions, which inhibits inhibitor of kappa light polypeptide gene enhancer in B-cells, kinase beta (IKBKB) [23]. Second, NF-кB activation occurs via inhibition of oxygen-dependent egl-9 family hypoxia-inducible factor 2 (EGLN2) that leads to CHUK activation and consequently IKBKB inhibition [23]. Third, NF-кB can be activated via tumor necrosis factor receptor superfamily, member 1A (TNFRSF1A) signaling, leading to TRAF6 activation and NF-кB induction [24]. The activation of NF-кB promotes various pathways directly and indirectly related to cell survival, including proliferation, inflammation, and survival.

As presented in Fig. 6a, treatment of SK-ChA-1 cells at low laser power resulted in upregulation of all members of the NF-кB subfamily. Strikingly, this response was not observed in the ITL 500 group, suggesting that the acute damage induced by the high-dose irradiation favored cell death signaling rather than cell salvage signaling. Furthermore, the ITL 50 group exhibited upregulated expression of NF-кB downstream genes (Table 4), including *CSF2*, *CXCL2*, and vascular cell adhesion molecule (*VCAM*). In addition, cells in the ITL 50 group also upregulated various pro-inflammatory cytokines, including IL1 alpha (*IL1A*), IL1 beta (*IL1B*), and *IL8*, with a log_2_ fold-change of 0.93, 1.48, and 6.75, respectively.

#### Activation of HIF-1 following PDT

Under normoxic conditions, HIF1A is hydroxylated by EGLN1 and hypoxia inducible factor 1, alpha subunit inhibitor (HIF1AN), which mediates recognition of HIF1A by von Hippel-Lindau tumor suppressor, E3 ubiquitin protein ligase (VHL) that targets HIF1A for ubiquitin-mediated proteasomal degradation. However, during oxidative stress and hypoxia, the oxygen sensors EGLN1 and HIF1AN are inhibited and thereby prevent degradation of HIF1A (reviewed in [25]). Downstream HIF-1 target genes then promote glycolysis, angiogenesis, and proliferation, all of which support cell survival. As shown in Fig. 6b, upregulation of HIF-1-induced genes was observed 90 min post-PDT in the ITL 50 group (Table 4). ZnPC-ITL-PDT led to upregulation of endothelin 1 (*EDN1*), a gene that is downstream of HIF1A and a known vasoconstrictor, in both groups after PDT. Unexpectedly, downregulation of vascular endothelial growth factor A (*VEGFA*), solute carrier family 2 (facilitated glucose transporter), member 1 (*SLC2A1*), and insulin-like growth factor binding protein 1 (*IGFBP1*) was observed in both groups.

## Discussion

Perihilar cholangiocarcinoma is a rare but highly lethal cancer that is typically diagnosed at an advanced tumor stage, accounting for the fact that the tumor cannot be resected in approximately 70–80 % of the patients [26]. It was demonstrated in a cohort of non-resectable patients that, when standard intervention (stenting) was combined with PDT, the median survival could be prolonged from 6 to 9 months to 21 months post-diagnosis (summarized in [27]). However, the management of non-resectable perihilar cholangiocarcinoma remains palliative. Inasmuch as PDT is a promising treatment strategy for perihilar cholangiocarcinoma, novel routes have been explored to increase therapeutic efficacy and to develop a more patient-friendly PDT strategy [11]. For those purposes, ZnPC-encapsulating liposomes (ITLs), which are part of a novel multi-targeted liposomal delivery platform for PDT [11, 14], were evaluated for toxicity and for the potential use in PDT of perihilar cholangiocarcinoma. The experiments demonstrated that (1) ZnPC-ITLs are not toxic *in vitro* and *in vivo* at high lipid concentrations, (2) irradiation of SK-ChA-1 cells at high laser power (500 mW, 15 J/cm^2^) resulted in more profound acute cell death than PDT at low laser power (50 mW, 15 J/cm^2^), and (3) irradiation of SK-ChA-1 cells at low laser power caused considerable survival signaling after PDT via activation of mainly HIF-1 and NF-кB.

The response of SK-ChA-1 cells to PDT at low (50 mW) or high laser power (500 mW) was compared. Since PDT treatment at low laser power causes moderate ROS production over an extended period of time [14], cells likely had the opportunity to activate an antioxidant (possibly via NFE2L2) and survival response to remediate the acute effects of ROS and cope with the ROS-induced damage more effectively than cells that were severely damaged by the 500-mW laser irradiation. This postulation is supported by the viability data, which demonstrated that cells irradiated at 50 mW were more viable at 90 min post-PDT than cells irradiated at 500 mW. The difference in cell viability at 90 min post-PDT was, however, abolished 24 h after PDT. A possible explanation is that, when cells are unable to cope with the PDT-inflicted damage, the execution of cell death programs via either apoptosis, (programmed) necrosis, and/or autophagy is ultimately completed. The time required to complete the activated cell death programs is apparently longer for moderately damaged cells than for highly damaged cells. Both PDT regimens also entirely abrogated metabolic activity 24 h post-PDT, which may be explained by the intracellular localization of ZnPC. ZnPC is largely confined to mitochondria upon cell entry [11], which is the source of electrons for the WST-1-based metabolic activity assay [28]. PDT-induced mitochondrial damage debilitates electron production and leakage from the electron transport chain, thereby hampering the reduction of WST-1 to the formazan chromophore. Metabolic perturbations (measured by WST-1) occur chronologically before cell death-mediated fragmentation and detachment from the wells plate (measured by the SRB assay), as a result of which the WST-1 data reflects more profound cell damage than the total protein assay at 24 h post-PDT.

To understand the molecular events that are triggered directly after PDT, whole genome expression profiles were established of PDT-treated SK-ChA-1 cells in the early phase (90 min) after PDT in line with previous reports [13]. Since the cellular redox state of a cell changes during PDT as a result of the production of ROS and reactive nitrogen species *(e.g.*, peroxynitrite) [29], PDT causes activation of a variety of redox-sensitive proteins and transcription factors [10]. ASK-1, also known as mitogen-activated protein kinase kinase kinase 5 (MAP3K5)) is associated with thioredoxin under physiological conditions and thereby kept inactive. However, during oxidative stress thioredoxin is oxidized and dissociates from ASK-1, leading to its activation [17]. Consequently, JUN N-terminal kinase (JNK, also designated as MAPK8) and protein 38 (p38) MAPK signaling is induced, leading to the immediate-early gene response via AP-1 [30]. Furthermore, UPR signaling and redox-sensitive transcription factors NFE2L2, NF-кB, and HIF1A are activated under oxidative stress and are able to initiate a plethora of processes (reviewed in [10]), including cell proliferation, inflammation, and angiogenesis.

When the PDT-treated groups were compared to the control group, SK-ChA-1 cells subjected to low-power PDT displayed a different response than cells treated using high-power PDT. Moreover, the number of up- and downregulated genes was considerably greater in the ITL 50 group than in the ITL 500 group. These data indicate that SK-ChA-1 cells treated by low-power PDT attempt to survive, as evidenced by the significant upregulation of HIF-1- (*P* < 0.001) and NF-κB-mediated (*P* < 0.001) pathways. It should be noted, however, that the experiments were carried out under normoxic conditions. Since HIF-1A is rapidly degraded under normoxia [31], the effects that were observed in terms of HIF-1 activation are probably an underestimation. Nevertheless, in line with the results of this study, Liu *et al*. observed significant upregulation of various proinflammatory genes, including *FOS*, *FOSB*, *IL8,* and tumor necrosis factor, alpha-induced protein 3 (*TNFAIP3*) in 5-aminolevulinic acid (5-ALA)-treated human gingival (Ca9-22) cells [32]. In our study, SK-ChA-1 cells treated with low-power PDT also demonstrated extensive upregulation of *FOS*, *FOSB*, *IL8*, and *TNFAIP3* (log_2_ fold-changes of 4.42, 2.13, 6.75, and 2.75, respectively). Moreover, Kammerer and co-workers observed significant upregulation of inflammation-related genes, including *CXCL2*, *CXCL3*, *IL1A*, and IL6 receptor (*IL6R*)) after non-lethal 5-ALA-PDT in a panel of prostate and glioblastoma cell lines [33]. Cells in the ITL 50 group also significantly upregulated *CXCL2* and *IL1A*, whereas *IL6R* remained unaffected. Contrary to expectations, cells in the ITL 50 group downregulated the NFE2L2-mediated pathway (*P* < 0.001); an effect that was absent in the ITL 500 group. This response could be due to the crosstalk between the NF-κB and NFE2L2 pathways. It has been proposed that RELA, a subunit of NF-κB, interferes with NFE2L2 activation through deprivation of CREB binding protein (CREBBP) and activation of histone deacetylase 3 (HDAC3) [34]. Thus, a strong induction of the NF-κB pathway, as observed in the ITL 50 group, may impede NFE2L2 signaling. Lastly, the UPR did not seem to be important in the early phase after PDT in SK-ChA-1 cells, which could be explained by the localization of ZnPC at the time of PDT. Since ZnPC translocates from the plasma membrane to intracellular organelles in a time-dependent manner [35], it is likely that the ZnPC concentration in the ER is low, as a result of which ER stress and the UPR were probably less important under these experimental conditions.

The data in this study as well as in previously published studies plead for the development and use of fourth-generation photosensitizers (*i.e.*, second-generation photosensitizer encapsulated in a nanoparticulate delivery system (making it a third-generation photosensitizer) with co-encapsulated small-molecular inhibitors of survival pathways) in PDT. In that respect, the HIF-1- and NF-κB-mediated survival responses that were induced by PDT in mainly the ITL 50 group comprise potential target sites for pharmacological intervention. Corroboratively, HIF-1 induction with cobalt chloride in human esophageal carcinoma (Het-1a) cells reduced the extent of cell death and abrogated apoptosis after 5-ALA-PDT [36]. This pro-survival response was blocked following HIF-1 silencing with siRNA, which augmented PDT efficacy in the Het-1a cells [36]. Chen *et al*. revealed that nanoparticulate delivery of HIF-1 siRNAs to head-and-neck carcinoma (SSC4) xenografts significantly enhanced photosan-PDT efficacy in mice [37], leading to 40 % tumor regression within 10 days post-PDT. Similarly, the combination treatment with ALA-PDT and celecoxib, an anti-inflammatory drug that inhibits prostaglandin-endoperoxide synthase 2 (PTGS2) (a downstream target of HIF-1 and NF-κB), yielded an additional 40 % reduction in tumor growth compared to ALA-PDT alone in human cholangiocarcinoma (HuCC-T1)-bearing mice [38]. Although the authors stated that increased ROS generation was mainly responsible for the increased response, it is likely that the inhibition of prostaglandin synthesis (reviewed in [39]), which is normally initiated by PTGS2 to promote survival, also contributed to therapeutic efficacy. Lastly, it was demonstrated that inhibition of HIF-1α with acriflavine, a small molecule that prevents the dimerization of HIF-1α with HIF-1β and thus its activation [40], potentiated PDT efficacy in human epidermoid carcinoma (A431) cells and SK-ChA-1 cells using liposomal ZnPC Broekgaarden *et al*., Inhibition of hypoxia inducible factor 1 with acriflavine sensitizes tumor cells to photodynamic therapy with zinc phthalocyanine-encapsulating cationic liposomes, in preparation [41].

Comparable results were obtained in studies where other survival pathways were inhibited before PDT. Coupienne *et al*. inhibited NF-кB with BAY 11–7082 (an inhibitor of IKK) prior to 5-ALA-PDT of glioblastoma cells [42], achieving increased therapeutic efficacy as a result of an impaired autophagic response, which otherwise mediates survival. Moreover, verteporfin-PDT induced epidermal growth factor receptor (EGFR) and signal transducer and activator of transcription 3 (STAT-3) signaling in ovarian carcinoma (OVCAR-5) and non-small cell lung cancer (H460) cells [43]. Activation of the STAT-3 pathway results in the transcription of both HIF-1- and NF-κB target genes [44, 45]. Accordingly, siRNA-mediated knockdown of either EGFR or STAT-3 increased PDT efficacy.

At this stage, combination treatments with respect to PDT and inhibitors in the clinical setting are limited to the treatment of macular degeneration, in which case VEGF inhibitors are employed to deter neovascularization. Nevertheless, the data that have become available to date indicate that the combined use of PDT and inhibitors of survival pathways in the form of fourth-generation photosensitizers may be an attractive approach to improve therapeutic efficacy.

## Conclusions

In summary, ZnPC-encapsulating liposomes are non-toxic in various *in vivo* models in the absence of irradiation but become highly cytotoxic upon PDT *in vitro*. Low-power PDT-treated perihilar cholangiocarcinoma cells activate extensive survival signaling *in vitro*, which is characterized by the induction of HIF-1- and NF-кB-related genes. Induction of these genes concurred with higher viability 90 min after PDT. Such post-PDT survival signaling may translate to a suboptimal therapeutic response in the clinical setting and possibly tumor recurrence. These findings encourage the development of photosensitizer delivery systems with co-encapsulated inhibitors of survival pathways, or so-called fourth-generation photosensitizers.

## Methods

### Chemicals

1,2-dipalmitoyl-*sn*-glycero-3-phosphocholine (DPPC) was obtained from Avanti Polar Lipids (Alabaster, AL). 1,2-distearoyl-*sn*-glycero-3-phosphoethanolamine-polyethylene glycol (DSPE-PEG, average PEG molecular mass of 2,000 amu), ZnPC (97 % purity), HEPES (4-(2-hydroxyethyl)-1-piperazineethanesulfonic acid), pyridine, and sulforhodamine B (SRB) were acquired from Sigma-Aldrich (St. Louis, MO). Acetic acid (glacial), ethidium bromide, ethylenediaminetetraacetic acid (EDTA), formaldehyde solution (36.5–38 % in water), sodium chloride, and tris(hydroxymethyl)aminomethane (Tris) were obtained from Merck KGaA (Darmstadt, Germany). Agarose was purchased from Gibco-BRL (Paisley, UK) and ethanol was from J.T. Baker (Deventer, the Netherlands).

All lipids were dissolved in chloroform and ZnPC was dissolved in pyridine at a 178-μM concentration. All dissolved lipids were stored under a nitrogen atmosphere at −20 °C.

### Cell culture

Human perihilar cholangiocarcinoma (SK-ChA-1) cells were maintained at standard culture conditions (37 °C, 5 % CO_2_ and 95 % air). SK-ChA-1 cells were cultured in Roswell Park Memorial Institute (RPMI) 1640 culture medium supplemented with 10 % fetal bovine serum (FBS) (v/v) (Gibco, Invitrogen, Carlsbad, CA), 1 % penicillin/streptomycin (v/v), 1 % L-glutamine (v/v) (both from Lonza, Walkersville, MD), and 1 × 10^−5^ % β-mercaptoethanol (v/v) (Sigma-Aldrich). The cells were passaged weekly at a 1:10 ratio.

### Preparation of ZnPC-ITLs

ZnPC-ITLs were prepared by the lipid film hydration technique as described previously [14]. Briefly, ZnPC-ITLs were composed of DPPC and DSPE-PEG (96:4, molar ratio). ZnPC was incorporated at a ZnPC-to-phospholipid ratio of 0.003. The liposomes were sized with a bath sonicator and characterized for size and polydispersity by photon correlation spectroscopy [14]. Liposome suspensions were purged with nitrogen gas and stored for a maximum of 3 days at 4 °C in the dark.

### PDT protocol

Cells were harvested using Accutase (Innovative Cell Technologies, San Diego, CA) and seeded in 6-wells culture plates (Corning Life Sciences, Tewksbury, MA) at a density of 0.5 × 10^6^ cells/well. After reaching confluence, cells were washed with PBS and incubated with ZnPC-ITLs (500 μM final lipid concentration) in serum-free RPMI 1640 medium (1.5 mL final volume per well) for 30 min at 37 °C in the dark. Control cells received an equal volume of PBS. In case of PDT, cells were irradiated with a 671-nm diode laser (CNI, Changchun, China) at a laser power of either 50 or 500 mW until a cumulative light dose of 15 J/cm^2^ was reached. PDT was performed in the dark while the cells were maintained at 37 °C using a hotplate (cat. no. 97042–616, VWR, Radnor, PA).

### Cell function and death assays

Mitochondrial metabolism was assessed using WST-1 reagent (Roche). Twenty-four hours post-PDT, the culture medium was removed and 1,500 μL of WST-1-containing RPMI medium (at a 1:25 volume ratio, serum- and phenol red-free) was added to the wells. After 30 min of incubation under standard culture conditions, 300-μL aliquots were transferred to 24-wells plates and the absorbance was read at 450 nm using 600 nm as a reference wavelength (BioTek Synergy HT multi-well plate reader, Winooski, VT). Data were normalized to the mean absorbance of the control cells.

In addition, cell death was determined 24 h post-PDT using the SRB total protein assay as described by Vichai *et al*. [46]. SRB absorbance was read at 564 nm using 690 nm as a reference wavelength (BioTek Synergy HT multi-well plate reader). Data were normalized to the mean absorbance of the control cells.

### Determination of mode of cell death

The mode of cell death following PDT was analyzed by flow cytometry using the Alexa Fluor 488 annexin V/dead cell apoptosis kit (Life Technologies, Carlsbad, CA). Cells were cultured in 6-wells plates as described in section "Cell culture" and irradiated as described in section "PDT protocol". Samples were prepared as described previously [14] and assayed on a FACSCanto II (Becton Dickinson, Franklin Lakes, NJ). Ten thousand events were recorded in the gated region and data were analyzed using FlowJo software (Treestar, Ashland, OR). Healthy cells were defined as annexin V-negative/propidium iodide (PI)-negative, apoptotic cells were defined as annexin V-positive/PI-negative, and necrotic cells were defined as PI-positive.

### ZnPC-ITL acute toxicity in chicken embryos

Fertilized chicken eggs (White Leghorn) were ordered from Drost Loosdrecht (Loosdrecht, the Netherlands), placed on paper towel in an egg incubator (Ova-Easy 190 Advance, Brinsea, Weston-super-Mare, UK), and maintained under dark conditions at 37.5 °C, 60 % humidity, and a 90° turn interval of 60 min. After 72 h, 2–3 mL of albumin was removed with a syringe to create empty volume in the superior portion of the egg. Next, surgical tape (Transpore White, 3M, St. Paul, MN) was fastened on the upper part of the eggshell and a small window (1.5 × 3.0 cm) was cut in the tape-covered eggshell that was immediately sealed with a second strip of surgical tape, after which the egg was placed back in the incubator. Previously opened eggs were incubated as described above but without the turn cycles. On embryonic development day 12, 50 μL of ZnPC-ITLs (final lipid concentration of 0.3, 0.5, or 0.7 mM in blood; blood volumes were derived from [47]) in 0.75 % NaCl (*i.e.*, iso-osmolar relative to embryonic blood) or 50 μL of 0.75 % NaCl (control) was intravenously injected into a large-sized blood vessel in the chorioallantoic membrane using a 30-gauge needle and a 1-mL syringe (Becton Dickinson). All surgical procedures were performed as fast as possible under sterile conditions in a LAF hood. Acute toxicity was defined as embryonic death within 24 h after systemic administration.

### ZnPC-ITL long term toxicity in mice

The animal experiments were approved by the animal ethics committee of the Academic Medical Center under BEX103077 and performed in accordance with the NIH *Guide for the Care and Use of Laboratory Animals*. Six-to-eight week old male C57BL/6 mice were purchased from Charles River (Leiden, the Netherlands). All mice were acclimated for 1 week and were provided with water and standard chow (Harlan Teklad, Harlan, Madison, WI) *ad libitum*. Mice were housed under green light at all times (Philips TL-D 36 W/17, Philips, Eindhoven, the Netherlands) with standard dark/light cycles to prevent activation of the photosensitizer. At the start of the experiment, mice were randomly assigned to the ZnPC-ITL or control group (*n* = 8 per group). A dose of 2.5 mM ZnPC-ITLs (final lipid concentration in blood, corresponding to an administered lipid dosage of 200 μmol/kg) was intravenously administered via the penile vein, whereas control mice received the same volume of buffer (0.88 % NaCl, 10 mM HEPES, pH = 7.4, 0.292 osmol/kg). Systemic lipid concentration was determined using a total blood volume of 80 mL/kg [48, 49]. Subsequently, all mice were inspected daily and were weighed every 4 days as part of toxicological vigilance. After 28 days, mice were anesthetized and blood was collected via cardiac puncture in heparin- or EDTA-anticoagulated microtainers (BD, Franklin Lakes, NJ). Biochemical and hematological parameters were determined by routine clinical chemistry (Department of Clinical Chemistry, Academic Medical Center). Lung, liver, and spleen tissue was loafed and fixed in FAA (47.5 % (v/v) ethanol, 5 % (v/v) acetic acid, 3.7 % (v/v) formaldehyde) at 4 °C, dehydrated in graded concentrations of ethanol and xylene, embedded in paraffin, cut to 5 μm-thick sections, and stained with hematoxylin and eosin as described in [50].

### Illumina HumanHT-12 array

SK-ChA-1 cells received either PBS (‘control’) or 500 μM ZnPC-ITLs (final lipid concentration) and were kept in the dark (‘ITL’), or were treated with 500-mW (‘ITL 500’) or 50-mW (‘ITL 50’) laser light (*n* = 3 per group). Ninety minutes after PDT, total cellular RNA was extracted from SK-ChA-1 cells using 1 mL of TRIzol (Life Technologies) according to the manufacturer’s protocol. RNA samples were purified using the RNeasy mini kit (Qiagen, Venlo, the Netherlands) and eluted in 30 μL RNAse-free H_2_O. The quality control, RNA labeling, hybridization, and data extraction were outsourced to ServiceXS (Leiden, the Netherlands). The RNA concentration was measured using a Nanodrop ND-1000 spectrophotometer (Nanodrop Technologies, Wilmington, DE) and the RNA quality and integrity was determined using Lab-on-Chip analysis on the Agilent BioAnalyzer (Agilent Technologies, Santa Clara, CA). Biotinylated cRNA was prepared using the Illumina TotalPrep RNA amplification kit (Ambion, Austin, TX) according to the manufacturer’s specifications with an input of 200 ng total RNA. Per sample, 750 ng of the obtained biotinylated cRNA samples was hybridized onto the Illumina HumanHT-12 v4 beadchip (Illumina, San Diego, CA). Hybridization and washing were performed according to the Illumina Manual “Direct Hybridization Assay Guide” and the scanning procedure was performed on the Illumina iScan (Illumina). Image analysis and extraction of raw expression data was performed with Illumina GenomeStudio v2011.1 Gene Expression software with default settings (no background subtraction and no normalization).

### Microarray preprocessing and data analysis

Analyses were carried out with Bioconductor packages using the statistical software package R (version 3.0.0). Raw data normalization was performed on the Illumina sample and control probe profiles by a normexp-by-control background correction, quantile normalization, and log_2_ transformation using the limma package (version 3.16.5). The arrayQualityMetrics package (version 3.16.0) was used to confirm that the microarray data was of good quality. Probes with a detection *P*-value of > 0.05 (non-expressed) on all arrays (17,521 of 47,231 probes) were filtered out. Principal component analysis was performed on unscaled data (function prcomp). Differential expression between the experimental conditions was assessed with a moderated t-test using the linear model framework from the limma package. Resulting *P*-values were corrected for multiple testing using the Benjamini-Hochberg false discovery rate. Corrected *P*-values of ≤ 0.05 were considered as statistically significant. Probes were reannotated using the Bioconductor package IlluminaHumanv4.db package (version 1.18.0). The microarray data have been deposited in NCBI Gene Expression Omnibus in a MIAME compliant format and are accessible under GEO series accession number GSE68292. In addition, a ROAST gene set test [51] was performed on the selected survival pathways (Additional file 3: Table S1) to statistically determine whether a survival pathway was upregulated or downregulated using 10,000 rotations with Benjamini-Hochberg-based multiple testing correction. If multiple probes were mapped to the same Entrez Gene identifier according to the illuminaHumanv4.db package, the probe with the highest standard deviation was chosen. Survival pathways were visualized using PathVisio 3.1.3. [52] on the basis of [10].

### Quantitative reverse transcription polymerase chain reaction (qRT*-*PCR)

The experiment as described in section "Illumina HumanHT-12 array" was repeated, RNA was extracted (section "Illumina HumanHT-12 array"), and cDNA was prepared using the oligo-dT-based Transcriptor first strand cDNA synthesis kit (Roche Diagnostics, Basel, Switzerland) with an input of 500 ng total RNA. In addition, 2 μM ribosomal protein S18 (RPS18) reverse transcriptase primer (GCATCGCCGGTCGGCATCG) was added to each reaction mix. cDNA was synthesized according to the manufacturer’s instructions and diluted in RNAse-free H_2_O to obtain a final concentration of 5 ng/μL.

For amplification reactions, 5 μL of 2× SensiFAST SYBR No-ROX master mix (Bioline, London, UK), 1 μL of forward and reverse primer mix (5 μM) (primer sequences can be found in Additional file 2: Figure S2), 2 μL of nuclease-free H_2_O, and 2 μL of cDNA template (10 ng) were mixed. The qRT-PCR reaction was carried out using a LightCycler 480 II instrument (Roche). The qRT-PCR program consisted of 3 min at 95 °C, 45 cycles of 1 s at 94 °C, 7 s at 65 °C, and 10 s at 72 °C, followed by melting curve analysis (65–97 °C, with a temperature increase of 0.11 °C/s). Subsequently, the quantitative analysis of the qRT-PCR data was performed according to Ruijter *et al*. [53] to calculate the starting concentration (N0) of each cDNA template. Gene expression levels of the target genes were normalized to the expression level of the reference gene *RPS18* and log_2_ fold-changes of the target genes were calculated based on the mean values of the control group.

To assure product specificity, all qRT-PCR amplification products were separated by gel electrophoresis using 2 % agarose in 0.5 × TAE buffer (40 mM Tris, 20 mM acetic acid, 1 mM EDTA) in the presence of 0.5 μg/mL ethidium bromide (Additional file 4: Figure S3). Subsequently, the ethidium bromide-stained qRT-PCR products were analyzed under UV using an ImageQuant LAS4000 imager (GE Healthcare Life Sciences, Piscataway, NJ). The O'GeneRuler DNA Ladder Mix (#SM1173, Thermo Scientific, Waltham, MA) was used as a reference to estimate the size of the qRT-PCR products. In addition, all qRT-PCR products were validated by sequencing. Therefore, qRT-PCR products were purified using the QIAquick PCR purification kit (Qiagen) and sequencing was performed using the BigDye Terminator Cycle sequencing kit (Thermo Scientific) according to the manufacturer’s instructions with an input of 5 ng DNA. Samples were sequenced on a Lifetech 3130xl genetic analyzer (Applied Biosystems, Waltham, MA) and data analysis was performed using NCBI BLAST (http://blast.ncbi.nlm.nih.gov/Blast.cgi). Sequencing results can be found in Additional file 5: Table S2.

### Statistical analysis

Statistical analysis was performed in GraphPad Prism 5 (GraphPad Software, La Jolla, CA). Normality was tested with the D’Agostino Pearson omnibus test. Differences between normally distributed variables were analyzed with an unpaired t-test or one-way ANOVA with Bonferroni post-hoc test and not normally distributed variables were analyzed with a Mann–Whitney U test. The Bonferroni method was applied to adjust the *P*-value in case of multiple testing (Additional file 1: Figure S1). Intergroup differences groups were indicated with (*) and differences between the treated groups and the control group were indicated with (#). A single, double, and triple sign indicate a *P*-value of ≤ 0.05, ≤ 0.01, and ≤ 0.001, respectively. Data are presented as mean ± SD throughout the manuscript.

## Additional files

Additional file 1: Figure S1.
*In vivo* toxicity evaluation of ZnPC-ITLs. (**A**) Chicken embryos were intravenously injected on embryonic development day 12 with different concentrations of ZnPC-ITLs (*n* = 7 per group). The concentrations indicate the final lipid concentration in blood. Control embryos were intravenously injected with an equal volume of 0.75 % NaCl. (**B**) C57BL/6 mice were intravenously injected with ZnPC-ITLs (2.5 mM, final lipid concentration in blood, red line) or physiological buffer (black line). Mice were weighed every four days until day 28 post-injection. Data is presented as mean ± SD with *n* = 8 per group. (**C**) Biochemical and hematological parameters assessed in C57BL/6 mice 28 days after systemic administration of ZnPC-ITLs. Data is presented as mean ± SD with *n* = 8 per group. Statistical analysis was performed as described in section "Statistical analysis". Abbreviations: ALT, alanine transaminase; AST, aspartate transaminase; CK-MB, creatine kinase M and B; CPK, creatine phosphokinase; LDH, lactate dehydrogenase. (**D**) Histology of liver, spleen, and lung of C57BL/6 mice 28 days after systemic administration of ZnPC-ITLs or physiological buffer (control). Hematoxylin and eosin staining, magnification 20 × . (DOC 5788 kb) Additional file 2: Figure S2. Validation of absolute log_2_ fold-changes as obtained by microarray with qRT-PCR. Microarray-derived transcript levels of a panel of genes are depicted for the ITL 50 and ITL 500 group in white and green, respectively. The corresponding qRT-PCR levels of these genes are depicted for the ITL 50 group in grey and the ITL 500 group in red. Gene expression is depicted as the log_2_ fold-change between treated and untreated cells. The microarray and qRT-PCR data were normalized to the expression level of the reference gene *RPS18*. (DOC 247 kb) Additional file 3: Table S1. Overview of transcriptional targets of the five survival pathways. Official gene names are listed that were derived from the Hugo Gene Nomenclature Committee (HGNC) along with their Genbank accession numbers (Gene ID). (XLSX 16 kb) Additional file 4: Figure S3. Analysis of qRT-PCR products by gel electrophoresis. (**A**) Lane 1 contains a ladder and lanes 2 – 8 show the specific qRT-PCR products of the genes that are listed on top of each lane. (**B**) Lane 1 contains a ladder and lane 2 shows the reference gene *RPS18*. The amplicon size of a specific gene product is noted in parentheses below each gene name. (DOC 1796 kb) Additional file 5: Table S2. Overview of primer sequences and resulting amplicon sizes (in bp) that were used for the validation of microarray expression levels using qRT-PCR. The primers are listed in the 5′ to 3′ direction. Primers were derived from RTPrimerDB (http://www.rtprimerdb.org/), NCBI primer Blast (http://www.ncbi.nlm.nih.gov/tools/primer-blast/), and qPrimerDepot (http://primerdepot.nci.nih.gov/). Sequencing of the qRT-PCR products, which were analyzed using NCBI Blast (http://blast.ncbi.nlm.nih.gov/Blast.cgi), confirmed the correct identity of each product. (XLSX 14 kb)

## Abbreviations

^1^O_2_: singlet oxygen
AP-1: activator protein 1
ARE: antioxidant responsive element
ARNT: aryl hydrocarbon receptor nuclear translocator
ASK-1: apoptosis signal regulating kinase 1
ATF3: activating transcription factor 3
BCL3: B-cell CLL/lymphoma 3
BMF: Bcl2 modifying factor
CCND1: cyclin D1
CDKN1B: cyclin-dependent kinase inhibitor 1B
CHUK: conserved helix-loop-helix ubiquitous kinase
CREBBP: CREB binding protein
CSF2: colony stimulating factor 2
CXCL1: chemokine (C-X-C motif) ligand 1
DPPC: 1,2-dipalmitoyl-sn-glycero-3-phosphocholine
DSPE-PEG: 1,2-distearoyl-sn-glycero-3-phosphoethanolamine-polyethylene glycol
DUSP6: dual specificity phosphatase 6
EDEM1: ER degradation enhancer, mannosidase alpha-like 1
EDN1: endothelin 1
EGFR: epidermal growth factor receptor
EDTA: ethylenediaminetetraacetic acid
EGLN2: oxygen-dependent egl-9 family hypoxia-inducible factor 2
EIF2AK3: eukaryotic translation initiation factor 2-alpha kinase 3
ER: endoplasmic reticulum
ERN1: endoplasmic reticulum to nucleus signaling 1
FBS: fetal bovine serum
FOS: FBJ murine osteosarcoma viral oncogene homolog
GCLC: glutamate-cysteine ligase, catalytic subunit
GCLM: glutamate-cysteine ligase, modifier subunit
GO: gene ontology
GSR: glutathione reductase
HBEGF: heparin-binding EGF-like growth factor
HDAC3: histone deacetylase 3
HEPES: (4-(2-hydroxyethyl)-1-piperazineethanesulfonic acid
HIF1A: hypoxia-inducible factor 1-alpha
HIF1AN: hypoxia inducible factor 1, alpha subunit inhibitor
HMOX1: heme oxygenase 1
HSPA1A: heat shock 70 kDa protein 1A
HSP90B1: heat shock protein 90 kDa beta
IGFBP1: insulin-like growth factor binding protein 1
IKBKB: inhibitor of kappa light polypeptide gene enhancer in B-cells, kinase beta
IL1A: IL1 alpha
IL1B: IL1 beta
IL6R: IL6 receptor
IL8: interleukin 8
ITLs: interstitially-targeted liposomes
JNK: JUN N-terminal kinase
JUNB: jun B proto-oncogen
KEAP1: Kelch-like ECH-associated protein 1
MAP: mitogen-activated protein
MAP3K5: mitogen-activated protein kinase kinase kinase 5
MAPK: MAP kinase
MMP1: matrix metallopeptidase 1
NFE2L2: nuclear factor (erythroid-derived 2)-like 2
NF-кB: nuclear factor of kappa light polypeptide gene enhancer in B-cells
NQO2: NAD(P)H dehydrogenase, quinone 2
ONOO-: peroxynitrite
p38: protein 38
PDT: photodynamic therapy
PEG: polyethylene glycol
PI: propidium iodide
PTGS2: prostaglandin-endoperoxide synthase 2
qRT-PCR: quantitative reverse transcription polymerase chain reaction
REL: v-rel avian reticuloendotheliosis viral oncogene homolog
ROS: reactive oxygen species
RPS18: ribosomal protein S18
SLC2A1: solute carrier family 2 (facilitated glucose transporter), member 1
SRB: sulforhodamine B
SRXN1: sulfiredoxin 1
STAT-3: signal transducer and activator of transcription 3
THBS1: thrombospondin 1
TNF: tumor necrosis factor
TNFAIP3: tumor necrosis factor, alpha-induced protein 3
TNFRSF1A: tumor necrosis factor receptor superfamily, member 1A
TRAF2: tumor necrosis factor receptor-associated factor 2
Tris: tris(hydroxymethyl)aminomethane
UPR: unfolded protein response
VCAM: vascular cell adhesion molecule
VEGFA: vascular endothelial growth factor A
VHL: von Hippel-Lindau tumor suppressor, E3 ubiquitin protein ligase
XBP1: X-box binding protein 1
ZnPC: zinc phthalocyanine
ZnPC-ITLs: ZnPC-encapsulating ITLs
