# Supplementary material for: Low-power photodynamic therapy induces survival signaling in perihilar cholangiocarcinoma cells
Source: BMC Cancer. 2015 Dec 26;15:1014. doi: 10.1186/s12885-015-1994-2 (PMC4691291; doi:10.1186/s12885-015-1994-2)
Supplement: Additional file 1: Figure S1. — In vivo toxicity evaluation of ZnPC-ITLs. (A) Chicken embryos were intravenously injected on embryonic development day 12 with different concentrations of ZnPC-ITLs (n = 7 per group). The concentrations indicate the final lipid concentration in blood. Control embryos were intravenously injected with an equal volume of 0.75 % NaCl. (B) C57BL/6 mice were intravenously injected with ZnPC-ITLs (2.5 mM, final lipid concentration in blood, red line) or physiological buffer (black line). Mice were weighed every four days until day 28 post-injection. Data is presented as mean ± SD with n = 8 per group. (C) Biochemical and hematological parameters assessed in C57BL/6 mice 28 days after systemic administration of ZnPC-ITLs. Data is presented as mean ± SD with n = 8 per group. Statistical analysis was performed as described in section "Statistical analysis". Abbreviations: ALT, alanine transaminase; AST, aspartate transaminase; CK-MB, creatine kinase M and B; CPK, creatine phosphokinase; LDH, lactate dehydrogenase. (D) Histology of liver, spleen, and lung of C57BL/6 mice 28 days after systemic administration of ZnPC-ITLs or physiological buffer (control). Hematoxylin and eosin staining, magnification 20 × . (DOC 5788 kb) [file 12885_2015_1994_MOESM1_ESM.doc]

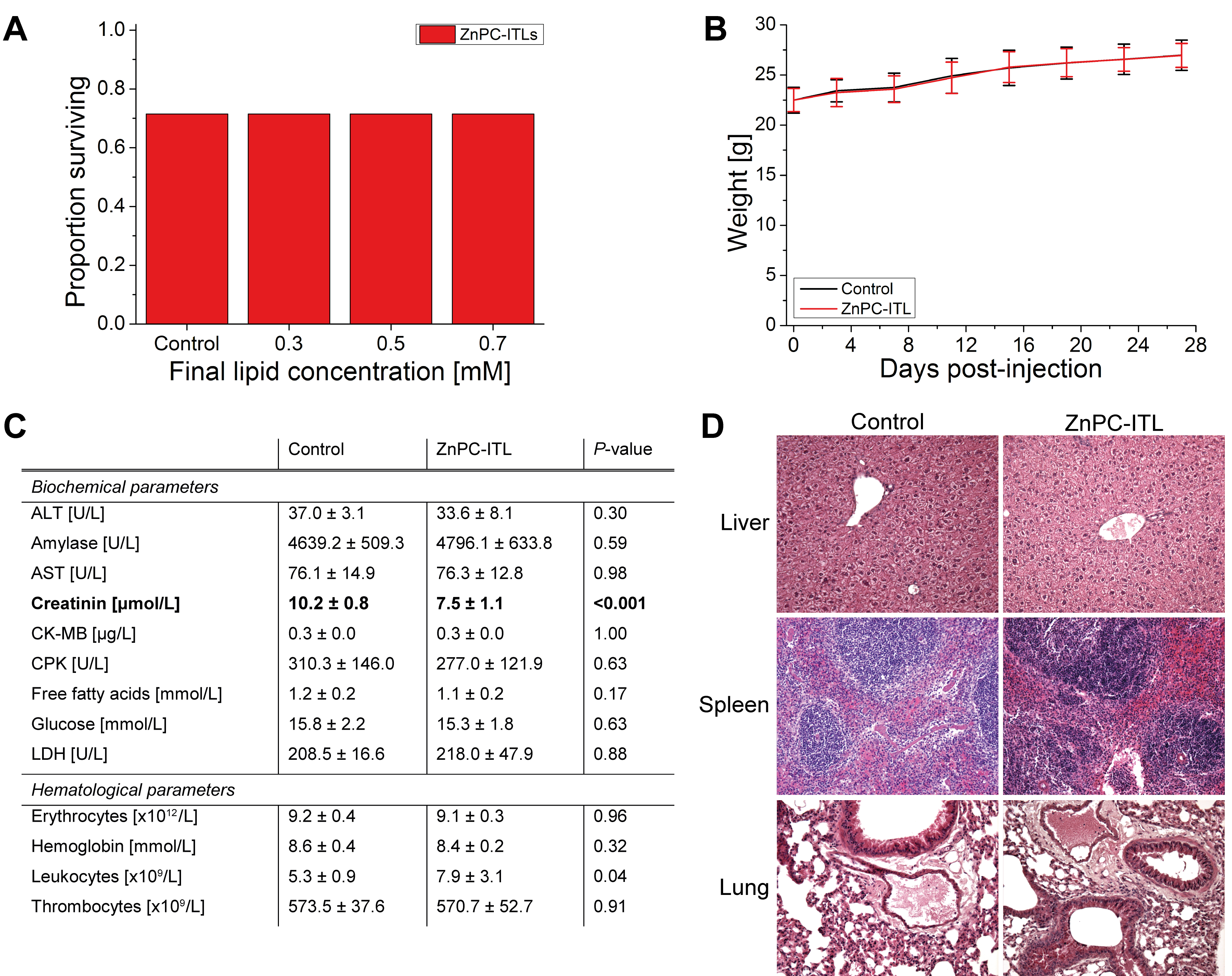
 **Supplementary Figure 1**. *In vivo* toxicity evaluation of ZnPC-ITLs. (A) Chicken embryos were intravenously injected on embryonic development day 12 with different concentrations of ZnPC-ITLs (n = 7 per group). The concentrations indicate the final lipid concentration in blood. Control embryos were intravenously injected with an equal volume of 0.75% NaCl. (B) C57BL/6 mice were intravenously injected with ZnPC-ITLs (2.5 mM, final lipid concentration in blood, red line) or physiological buffer (black line). Mice were weighed every four days until day 28 post-injection. Data is presented as mean ± SD with n = 8 per group. (C) Biochemical and hematological parameters assessed in C57BL/6 mice 28 days after systemic administration of ZnPC-ITLs. Data is presented as mean ± SD with n = 8 per group. Statistical analysis was performed as described in section 5.12. Abbreviations: ALT, alanine transaminase; AST, aspartate transaminase; CK-MB, creatine kinase M and B; CPK, creatine phosphokinase; LDH, lactate dehydrogenase. (D) Histology of liver, spleen, and lung of C57BL/6 mice 28 days after systemic administration of ZnPC-ITLs or physiological buffer (control). Hematoxylin and eosin staining, magnification 20×.
