# Supplementary material for: Low-power photodynamic therapy induces survival signaling in perihilar cholangiocarcinoma cells
Source: BMC Cancer. 2015 Dec 26;15:1014. doi: 10.1186/s12885-015-1994-2 (PMC4691291; doi:10.1186/s12885-015-1994-2)
Supplement: Additional file 2: Figure S2. — Validation of absolute log2 fold-changes as obtained by microarray with qRT-PCR. Microarray-derived transcript levels of a panel of genes are depicted for the ITL 50 and ITL 500 group in white and green, respectively. The corresponding qRT-PCR levels of these genes are depicted for the ITL 50 group in grey and the ITL 500 group in red. Gene expression is depicted as the log2 fold-change between treated and untreated cells. The microarray and qRT-PCR data were normalized to the expression level of the reference gene RPS18. (DOC 247 kb) [file 12885_2015_1994_MOESM2_ESM.doc]

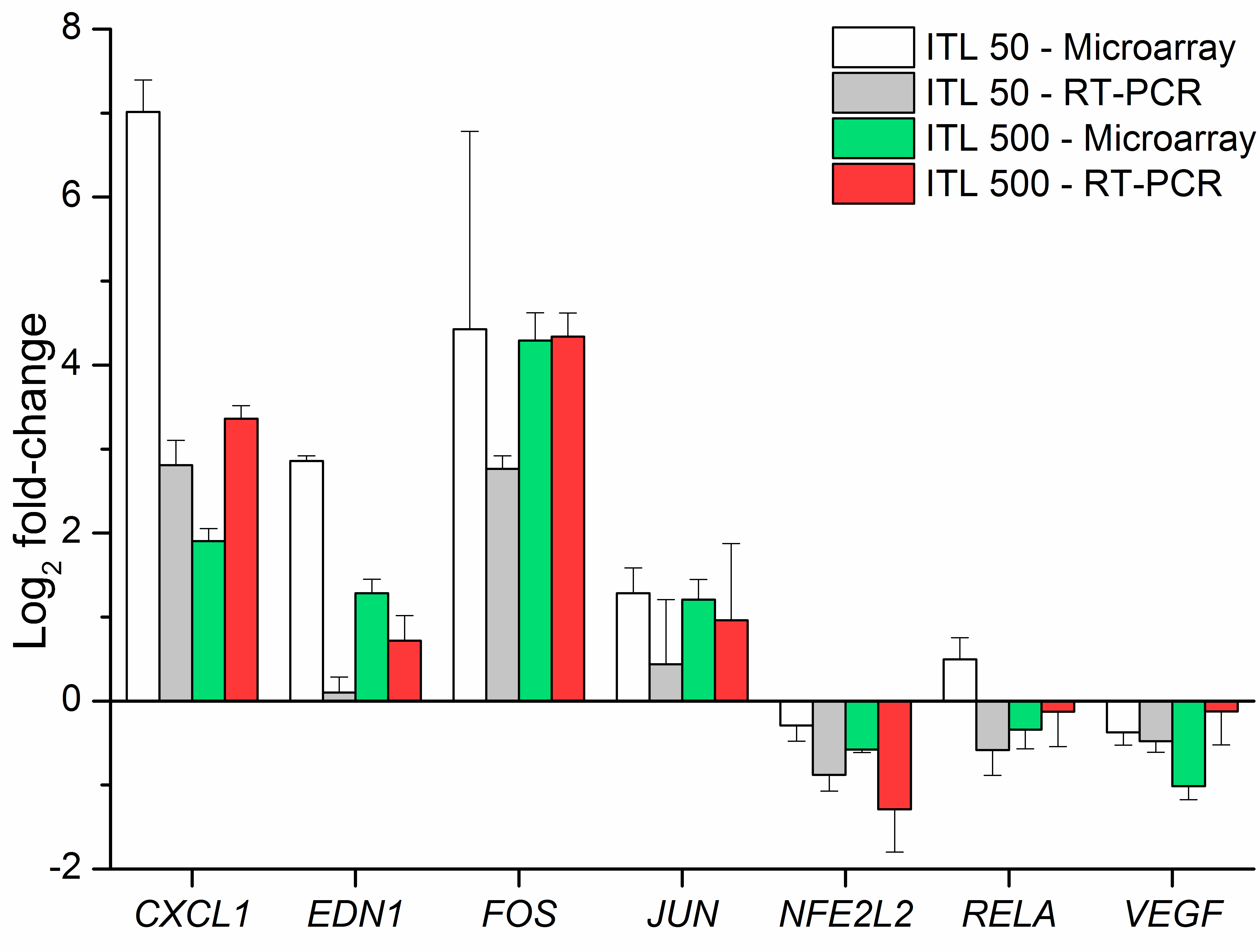


**Supplementary Figure 2**. Validation of absolute log_2_ fold-changes as obtained by microarray with qRT-PCR. Microarray-derived transcript levels of a panel of genes are depicted for the ITL 50 and ITL 500 group in white and green, respectively. The corresponding qRT-PCR levels of these genes are depicted for the ITL 50 group in grey and the ITL 500 group in red. Gene expression is depicted as the log_2_ fold-change between treated and untreated cells. The microarray and qRT-PCR data were normalized to the expression level of the reference gene *RPS18*.
