# Supplementary material for: Low-power photodynamic therapy induces survival signaling in perihilar cholangiocarcinoma cells
Source: BMC Cancer. 2015 Dec 26;15:1014. doi: 10.1186/s12885-015-1994-2 (PMC4691291; doi:10.1186/s12885-015-1994-2)
Supplement: Additional file 4: Figure S3. — Analysis of qRT-PCR products by gel electrophoresis. (A) Lane 1 contains a ladder and lanes 2 – 8 show the specific qRT-PCR products of the genes that are listed on top of each lane. (B) Lane 1 contains a ladder and lane 2 shows the reference gene RPS18. The amplicon size of a specific gene product is noted in parentheses below each gene name. (DOC 1796 kb) [file 12885_2015_1994_MOESM4_ESM.doc]

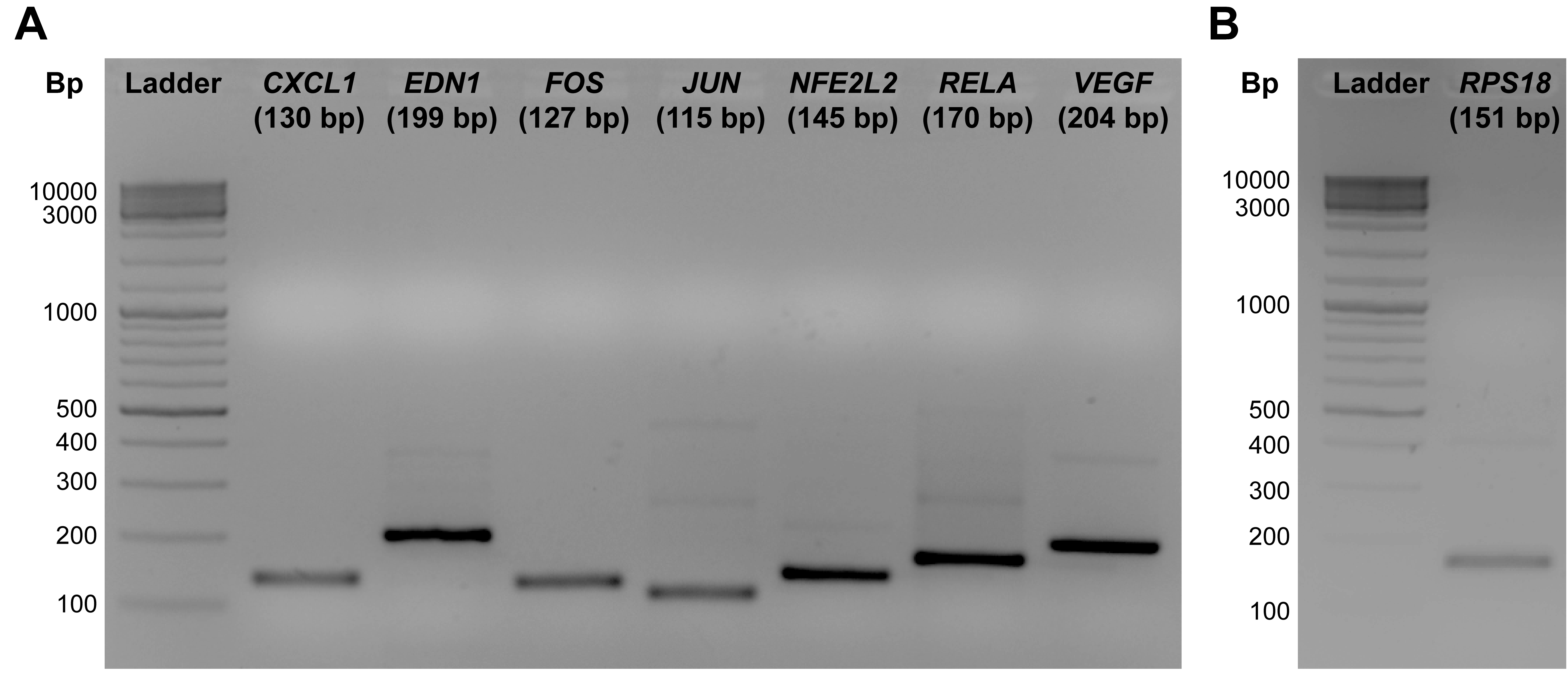


**Supplementary Figure 3**. Analysis of qRT-PCR products by gel electrophoresis. (A) Lane 1 contains a ladder and lanes 2 – 8 show the specific qRT-PCR products of the genes that are listed on top of each lane. (B) Lane 1 contains a ladder and lane 2 shows the reference gene *RPS18*. The amplicon size of a specific gene product is noted in parentheses below each gene name.
